# Global trends and disparities in burden of blindness and vision loss caused by non-communicable diseases from 1990 to 2021, and forecasts to 2045: a systematic analysis for the global burden of disease study 2021

**DOI:** 10.3389/fmed.2025.1561568

**Published:** 2025-06-19

**Authors:** Lingxia Ye, Xin Huang, Yufeng Xu

**Affiliations:** ^1^Department of Endocrinology and Metabolism, The Second Affiliated Hospital of Zhejiang University School of Medicine, Hangzhou, China; ^2^Department of Endocrinology and Metabolism, The Fifth Affiliated Hospital of Wenzhou Medical University, Lishui Municipal Central Hospital, Lishui, China; ^3^Department of Ophthalmology, The Second Affiliated Hospital of Zhejiang University School of Medicine, Hangzhou, China

**Keywords:** blindness and vision loss, non-communicable diseases, burden of disease, years lived with disability, disparity

## Abstract

**Background:**

Blindness and vision loss (BVL) is a major public health concern. Non-communicable diseases (NCDs), including cataract, glaucoma, age-related macular degeneration, diabetic retinopathy and etc., were the leading causes of vision impairment globally.

**Methods:**

We extracted global, regional, national, age-and sex-specific data on the prevalence and years lived with disability (YLDs) of BVL caused by NCDs from the Global Burden of Disease Study 2021 (GBD 2021), and then conducted a secondary comparative analysis based on time, location, age, gender, socioeconomic development index (SDI) and health system level.

**Results:**

From 1990 to 2021, the global incidence of BVL caused by NCDs continuously increased. In 2021, 1475648.6 thousand BVL cases caused by NCDs occurred globally, and ASR of YLDs reached 371.1 per 100,000 population. Great disparities were found across different genders, ages, and locations. Higher burdens were noted among females, older adult individuals, regions with lower SDI or less advanced health systems.

**Conclusion:**

The burden of BVLs caused by NCDs has increased significantly since 1990 and varies widely across regions. Greater efforts are needed in NCDs control and vision protection, especially in older adult individuals and females, in regions with lower SDI, and in regions with less advanced health systems.

## Introduction

Blindness constitutes a significant public health and socioeconomic challenge, impacting not merely economic and educational prospects, but also diminishing the quality of life and elevating the mortality risk (1, 2). In 2020, an estimated 43·3 million people were blind, and an estimated 295 million people suffered from moderate and severe vision loss (3). To combat this issue, the World Health Organization (WHO) launched the “VISION 2020: The Right to Sight” (4), a collaborative global effort aimed at eradicating avoidable blindness, thereby mobilizing systematic actions to eliminate preventable causes of blindness. However, despite substantial efforts in vision protection, the burden of vision impairment due to age-related eye diseases, diabetic retinopathy, and other chronic eye conditions may continue to rise, driven by population aging and the increasing prevalence of obesity and diabetes (5, 6).

Concurrently, as socio-economic progress advances and life expectancy extends, the disease burden is undergoing a significant shift from communicable diseases to non-communicable diseases (NCDs) and age-related conditions (7, 8). Uncorrected refractive error, cataract, age-related macular degeneration, glaucoma, and diabetic retinopathy have emerged as the leading global causes of vision impairment (9), all of which are quintessential NCDs (10). Consequently, BVL resulting from NCDs have become pivotal targets for vision protection initiatives. Notably, a substantial proportion of BVLs attributed to NCDs are avoidable, especially refractive errors and cataracts (9). Hence, it is of paramount importance to delve into the burden of BVL caused by NCDs and to shine a spotlight on the disparities that exist. Regrettably, there is a paucity of reports examining the disparities in the burden of BVL caused by NCDs at the global, national, socioeconomic, gender, and age-specific levels.

To bridge this gap, the present study aimed to conduct a comprehensive assessment of the burden of BVL attributable to NCDs, examining it across various dimensions including time, nation, age, gender, socioeconomic status, and health system level and forecast this burden up to the year 2045. The overarching goal is to provide crucial insights and data that can inform the development of targeted health policies, thereby aiding in the formulation of more effective strategies to address the significant public health challenge posed by BVL due to NCDs.

## Methods

### Study design and data sources

This study was a secondary analysis based on open database. Ethical approval was not applicable for this study. Data on the prevalence and years lived with disability (YLDs) of blindness and vision loss were obtained from the Global Burden of Disease study 2021 (GBD 2021), which was collected from the Global Health Data Exchange database.^1^ GBD 2021 study encompassed individuals of all ages and genders across the globe. The study estimates are given for 371 diseases and injuries; 204 countries and territories; 25 age groups; females, males, and both sexes combined; and for the years 1990–2021 (10).

### Case definition

In GBD 2021 study, vision loss was estimated, and categorized according to the severity of disease as follows: (1) blindness, distance visual acuity of <3/60 or <10% visual field around central fixation; (2) severe vision loss, distance visual acuity ≥3/60 and <6/60; (3) moderate vision loss, distance visual acuity ≥6/60 and <6/18; and (4) near vision loss (uncorrected presbyopia), near visual acuity of <6/12 distance equivalent.

In GBD 2021 study, all causes were aggregated into three groups: communicable, maternal, neonatal, and nutritional diseases (Group 1 diseases); non-communicable diseases (Group 2); and injuries (Group 3). The non-communicable diseases group mainly include neoplasms, cardiovascular diseases, chronic respiratory diseases, digestive diseases, neurological disorders, mental disorders, substance use disorders, diabetes and kidney diseases, skin and subcutaneous diseases, sense organ diseases, musculoskeletal disorders, and other non-communicable diseases. Sense organ diseases included BVL, age-related and other hearing loss and other sense organ diseases.

YLDs were calculated with a microsimulation process that used estimated age-sex-location-year-specific prevalent counts of nonfatal disease sequelae (consequences of a disease or injury) for each cause and disability weights for each sequela as the inputs (10). The sociodemographic index (SDI) was applied as a composite indicator of social and economic conditions. It is the geometric mean of 0 to 1 indices of the fertility rate among females younger than 25 years, average years of education for those aged 15 years or older and lagged distributed income per capita (10), ranging from 0 to 1. These quintiles include low SDI (<0.45), low-middle SDI (≥0.45 and <0.60), middle SDI (≥0.60 and <0.69), high-middle SDI (≥0.69 and <0.805), and high SDI(≥0.80).

### Statistical analysis

The data are presented as values with 95% uncertainty intervals (UIs). The age-standardized rates of YLDs were expressed as the number per 100,000 population. The Kruskal-Wallis test was used with non-normal distributions to evaluate the difference in age-standardized rates between males and females. The autoregressive integrated moving average (ARIMA) model, widely used in time series analysis (11, 12), was applied to estimate the burden of vision loss attributable to diabetes from 2021 to 2045 (R system, version 4.2.2; detailed method in the Supplementary File 2). Most statistical analyses, except those specified above, were conducted via Prism software version 9.0 (GraphPad, San Diego, California). A *p* value less than 0.05 was considered statistically significant.

## Results

### Global burden of BVL caused by NCDs from 1990 to 2021

Globally, in 2021, the number of BVL cases caused by NCDs reached 1475648.6 (95% UI: 1193586.9–1821128.1) thousand, with an increasement of 147.4% compared to that in 1990 [596532.2 (95% UI: 491641.5–726373.0) thousand], and the age-standardized rate (ASR) was 17.2 (95% UI: 14.0–21.2) thousand per 100,000 population in 2021, with an increasement of 24.6% compared to that in 1990 [13.8 (95% UI: 11.3–16.7) thousand per 100,000 population] (Table 1). The prevalence of YLDs in 1990 was 15379.4 (95% UI: 10402.7–22127.3) thousand, increasing to 31573.5 (95% UI: 20473.3–46732.3) thousand (Table 1). The ASR of YLDs in 1990 was 370.1 (95% UI: 252.5–534.0) per 100,000 population, and increased to 371.1 (95% UI: 241.2–547.7) in 2021.

**Table 1:** Prevalence of blindness and vision loss caused by NCDs in 1990 and 2021 by GBD 2021 super regions.

GBD 2021 Super Regions	All-ages number		Age-standardized rate		AAPC of number (%)	AAPC of rate (%)
	(×1,000, 95%UI)		(/100,000, 95% UI)			
	1990	2021	1990	2021		
Global	596532.2 (491641.5,726373.0)	1475648.6 (1193586.9,1821128.1)	13759.7 (11333.1,16713.9)	17243.1 (14032.7,21189.8)	4.75	0.82
Gender						
Male	261169.1 (215432.8, 319597.2)	654779.6 (523202.0, 818773.9)	12595.8 (10385.2, 15335.1)	15789.6 (12708.7, 19586.9)	4.86	0.82
Female	335363.2 (275686.1,408257.7)	820869.0 (670001.3, 1002533.5)	14865.6 (12224.0, 18119.9)	18629.7 (15227.6, 22772.8)	4.67	0.82
SDI grouping levels						
High SDI	85415.5 (67749.0, 107172.5)	147302.6 (117053.1, 184223.1)	8403.5 (6721.2, 10547.8)	8988.8 (7162.1, 11295.5)	2.34	0.22
High-middle SDI	128761.5 (104369.9, 158957.6)	293840.1 (232287.4,365492.5)	12666.2 (10354.8, 15498.5)	16023.5 (12813.1, 19915.2)	4.14	0.86
Middle SDI	189972.3 (156329.8, 230449.5)	542760.7 (435049.0,673035.3)	15766.8 (13023.8, 19047.8)	19740.9 (16068.2, 24287.4)	5.99	0.81
Low-middle SDI	141073.9 (117212.3, 170511.5)	357454.7 (291728.3, 435253.7)	19680.7 (16530.1, 23486.3)	22207.8 (18295.8, 26613.8)	4.95	0.41
Low SDI	50806.2 (41758.5, 62944.4)	133440.1 (108404.7, 164636.2)	18159.1 (15012.3, 21995.5)	20168.2 (16743.2, 24196.8)	5.25	0.36
Health system grouping levels						
Advanced Health System	146948.8 (118289.5, 183865.8)	226864.1 (181432.3, 282001.7)	9629.9 (7797.1, 11916.9)	9916.5 (7959.6, 12363.5)	1.75	0.10
Basic Health System	245188.6 (200626.5, 298486.3)	661098.3 (523023.5, 825577.8)	14491.4 (11899.0, 17629.2)	17613.4 (14173.2, 21767.5)	5.47	0.69
Limited Health System	191013.2 (157276.5, 232039.8)	555557.1 (450579.0,678692.4)	20401.8 (17062.3, 24452.9)	24403.2 (20088.1, 29187.9)	6.16	0.63
Minimal Health System	12878.8 (10279.5, 16391.4)	31278.8 (24932.1, 39980.2)	16522.6 (13349.2, 20416.5)	16939.6 (13693.0, 20721.7)	4.61	0.08
High income						
High-income Asia Pacific	17289.2 (13419.3, 22589.4)	27079.2 (21305.8, 34298.3)	8685.9 (6822.9, 11152.9)	8515.6 (6718.3, 10996.4)	1.83	−0.06
Western Europe	42894.8 (34620.2, 53985.4)	59921.2 (47722.6, 75487.7)	8518.5 (6905.7, 10572.3)	8325.4 (6746.8, 10334.1)	1.28	−0.07
Australasia	1853.2 (1462.3, 2368.0)	3537.8 (2789.3, 4455.0)	8447.8 (6696.3, 10862.9)	8390.8 (6673.4, 10680.7)	2.93	−0.02
High-income North America	21587.0 (17059.2, 27346.4)	37633.5 (29242.7, 48013.4)	6932.4 (5463.2, 8884.4)	7447.0 (5743.2, 9799.6)	2.40	0.24
Southern Latin America	4612.9 (3758.5, 5715.2)	7679.3 (6224.3, 9627.6)	9816.0 (8016.6, 12215.0)	9632.6 (7834.0, 12005.5)	2.14	−0.06
Central Europe, Eastern Europe and Central Asia						
Central Europe	11986.3 (9656.4, 15006.7)	15752.6 (12493.5, 20076.2)	8423.8 (6831.5, 10487.7)	8292.8 (6694.3, 10297.8)	1.01	−0.05
Eastern Europe	40482.7 (32576.4, 50075.1)	54176.4 (43253.7, 66887.6)	15184.2 (12304.5, 18538.0)	17503.0 (14087.9, 21602.8)	1.09	0.49
Central Asia	6605.7 (5602.7, 7861.7)	10723.9 (8815.4, 13055.7)	13097.0 (11049.1, 15727.6)	12633.1 (10426.7, 15447.3)	2.01	−0.11
Latin America and Caribbean						
Tropical Latin America	21726.7 (17667.3, 26972.6)	47862.8 (38518.9, 59715.7)	19313.6 (15752.9, 23892.0)	18818.9 (15276.5, 23234.6)	3.88	−0.08
Central Latin America	15887.2 (13492.2, 18687.6)	40715.8 (33085.3, 50123.0)	14858.3 (12590.0,17527.6)	15846.1 (12914.3, 19420.2)	5.04	0.21
Andean Latin America	4708.9 (3860.4, 5773.5)	11335.1 (9154.7, 14019.7)	18900.5 (15454.8,23143.0)	18155.4 (14719.4, 22251.9)	4.54	−0.13
Caribbean	4256.6 (3478.0,5213.5)	7578.1 (6106.1,9437.8)	14852.6 (12144.5,18226.8)	14483.2 (11686.2, 17929.5)	2.52	−0.08
Southeast Asia, East Asia, and Oceania						
East Asia	126285.6 (100738.7, 158108.4)	393370.8 (304434.7, 505808.7)	13216.6 (10730.3, 16505.6)	18520.4 (14491.0, 23383.0)	6.82	1.29
Southeast Asia	45712.5 (38140.3, 55029.7)	103179.4 (84460.3, 127050.3)	15185.4 (12724.3, 17979.5)	15094.9 (12491.0, 18188.1)	4.06	−0.02
Oceania	578.1 (476.3, 709.6)	1416.6 (1157.7, 1770.0)	16038.3 (13316.6, 19527.9)	15787.0 (13084.8, 19254.9)	4.68	−0.05
North Africa and Middle East						
North Africa and Middle East	31776.4 (27147.1, 36760.7)	79320.6 (65144.2, 97892.6)	15177.6 (12862.5, 17770.6)	15367.3 (12744.9, 18568.8)	4.83	0.04
South Asia						
South Asia	148369.4 (122260.2, 180639.1)	455665.4 (365608.4, 555727.5)	22121.2 (18548.2, 26456.3)	27696.8 (22558.4, 33326.6)	6.68	0.81
Sub-Saharan Africa						
Southern Sub-Saharan Africa	9950.2 (7806.8, 12735.5)	20066.0 (15775.0, 25305.2)	27668.0 (22088.4, 34469.3)	28206.2(22796.7, 34718.1)	3.28	0.06
Eastern Sub-Saharan Africa	14072.3 (11323.6, 17416.1)	33072.4 (26497.4, 41730.9)	14320.4 (11651.1, 17479.3)	14194.0 (11507.2, 17358.6)	4.36	−0.03
Central Sub-Saharan Africa	6724.0 (5147.7, 8776.2)	17454.3 (13258.9,23163.3)	20880.4 (16479.9, 26588.0)	20778.6 (16297.2, 26617.3)	5.15	−0.02
Western Sub-Saharan Africa	19172.5 (15321.9, 24230.2)	48107.3 (38498.5, 61312.9)	17206.1 (13931.0, 21431.5)	17509.6 (14320.1, 21592.4)	4.87	0.06

YLDs, years lived with disability; NCDs, non-communicable diseases; AAPC, average annual percent of change.

**Figure 1:**
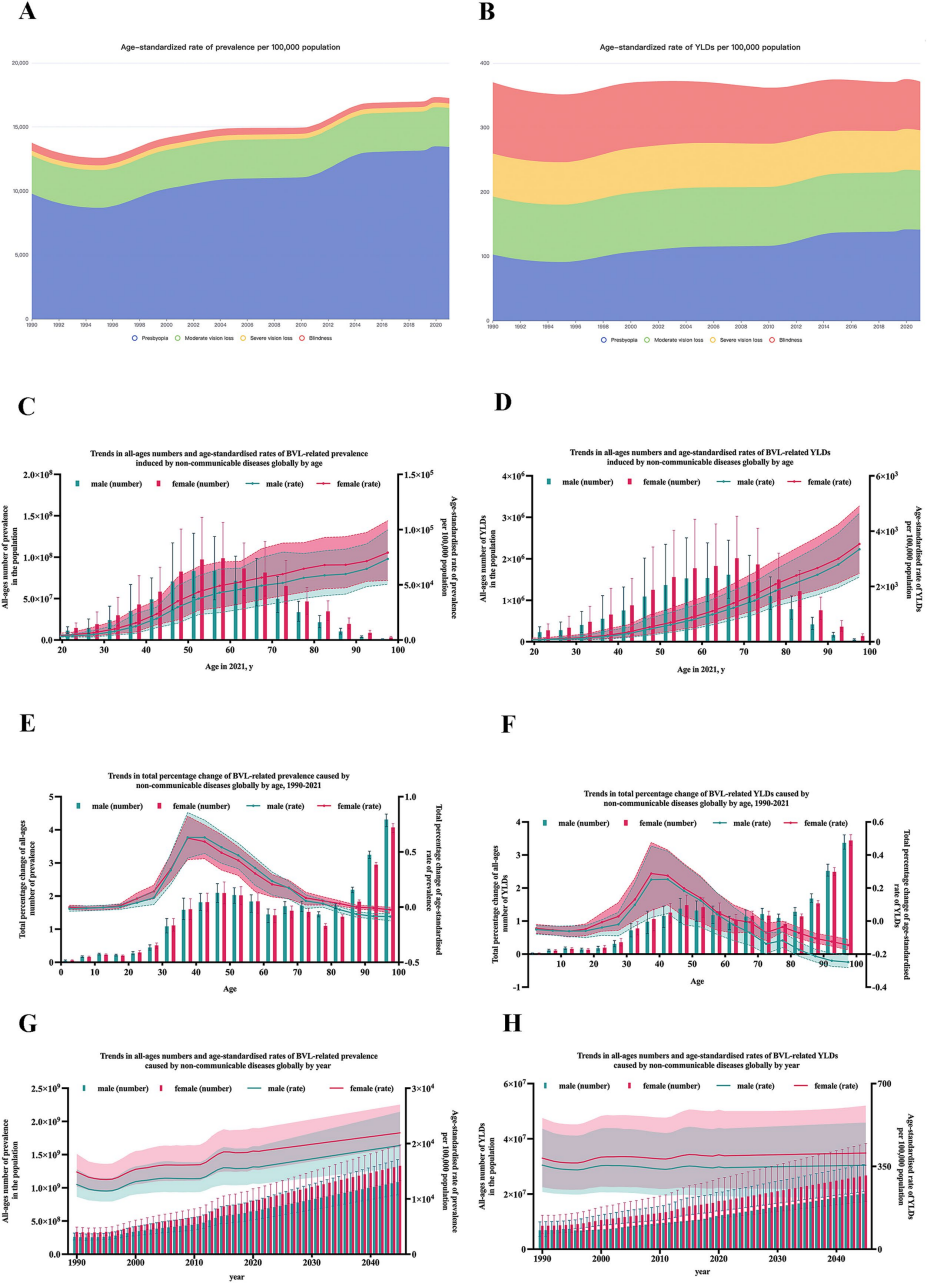
Prevalence and YLDs of blindness and vision loss caused by NCDs globally. **(A)** ASR of prevalence of BVL caused by NCDs by severity from 1990 to 2021; **(B)** ASR of YLDs of BVL caused by NCDs by severity from 1990 to 2021; **(C)** trends in total percentage change of prevalence of BVL caused by NCDs by gender and age; **(D)** trends in total percentage change of YLDs of BVL caused by NCDs by gender and age; **(E)** trends in total percent change of prevalence by gender and age; **(F)** trends in total percent change of YLDs by gender and age; **(G)** trends in prevalence of BVL caused by NCDs by gender and year; **(H)** trends in YLDs of BVL caused by NCDs by gender and year. YLDs, years lived with disability; NCDs, non-communicable diseases; BVL, blindness and vision loss; ASR, age-standardized rate.

According to severity, BVL was divided into four levels. In 2021, the prevalence number of BVL caused by NCDs included presbyopia [1155063.1 (95%UI: 875226.1–1514597.5) thousand], moderate vision loss [255093.5 (95%UI: 228145.3–284942.7) thousand], severe vision loss [29963.5 (95%UI: 25821.1–34281.5) thousand] and blindness [35528.5 (95%UI: 31362.6–40245.6) thousand] (Table 1 and Supplementary Table 1). And ASR of prevalence per 100 thousand population varied in different severity levels: presbyopia [13436.2 (95%UI: 10223.5–17585.8)], moderate vision loss [3034.6 (95%UI: 2721.2–3370.5)], severe vision loss [352.3 (95%UI: 304.7–402.0)] and blindness [420.0 (95%UI: 304.7–474.3)] (Figures 1A,B). In addition, the number of YLDs [presbyopia: 12152.8 (95%UI: 5363.1–23487.2) thousand; moderate vision loss: 7725.8 (95%UI: 4746.8–12016.3) thousand; severe vision loss: 5302.4 (95%UI: 3701.4–7627.2) thousand; blindness: 6392.4 (95%UI: 4328.1–8901.1) thousand] also varied by severity. Compared to the ASR of YLDs in 1990 [presbyopia: 102.8 (95%UI: 45.6–201.1); moderate vision loss: 90.0 (95%UI: 55.5–140.3); severe vision loss: 66.7 (95%UI: 46.5–96.0); blindness: 110.6 (95%UI: 75.3–153.4)], ASR of YLDs of presbyopia [141.3 (95%UI: 62.5–272.7)] and moderate vision loss [91.9 (95%UI: 56.6–143.1)] both increased in 2021, while severe vision loss [62.3 (95%UI: 43.6–90.0)] and blindness [75.5 (95%UI: 51.2–104.7)] both decreased in 2021.

### Burden of BVL caused by NCDs by age and gender

With age, the burden of BVL caused by NCDs showed great disparity, as shown in Figures 1C–F. The peak of prevalence number arrived at age 55–59 years for both females [98795.0 (95%UI: 58957.5–142016.6) thousand] and males [83479.2 (95%UI: 48478.0–125047.4) thousand], and the peak of YLDs number all arrived at age 65–69 years [female: 2013.7 (95%UI: 1320.2–3025.2) thousand; male: 1616.2 (95%UI: 1049.0–2447.4) thousand]. After standardized for age, the prevalence rate and YLDs rate all gradually increased with age for both female and male.

To observe the growth rate in different age groups, the percent change in burden was calculated to represent the average annual rate of growth from 1990 to 2021. The most rapid growth of prevalence rate presented at age 35–39 years for females [28.8% (95%UI: 6.4–47.5%)] and at age 40–44 years for males [25.3% (95%UI: 4.6–42.3%)]. And the most rapid growth of YLDs rate all presented at age 35–39 years [female: 63.2% (95%UI: 44.5–85.6%); male: 62.5% (95%UI: 43.2–82.3%)].

For gender disparity, the burden of BVL caused by NCDs in females remained consistently heavier than those in males in all age groups in 2021, as shown in Figures 1C,D. However, the trends varied in different age groups. The growth rate of prevalence rate was significantly higher in males at the 35–66 years of age, although the growth rate of YLDs rate was invariably higher in females in all age groups, as shown in Figures 1E,F.

### Burden of BVL caused by NCDs by country and territory

**Figure 2:**
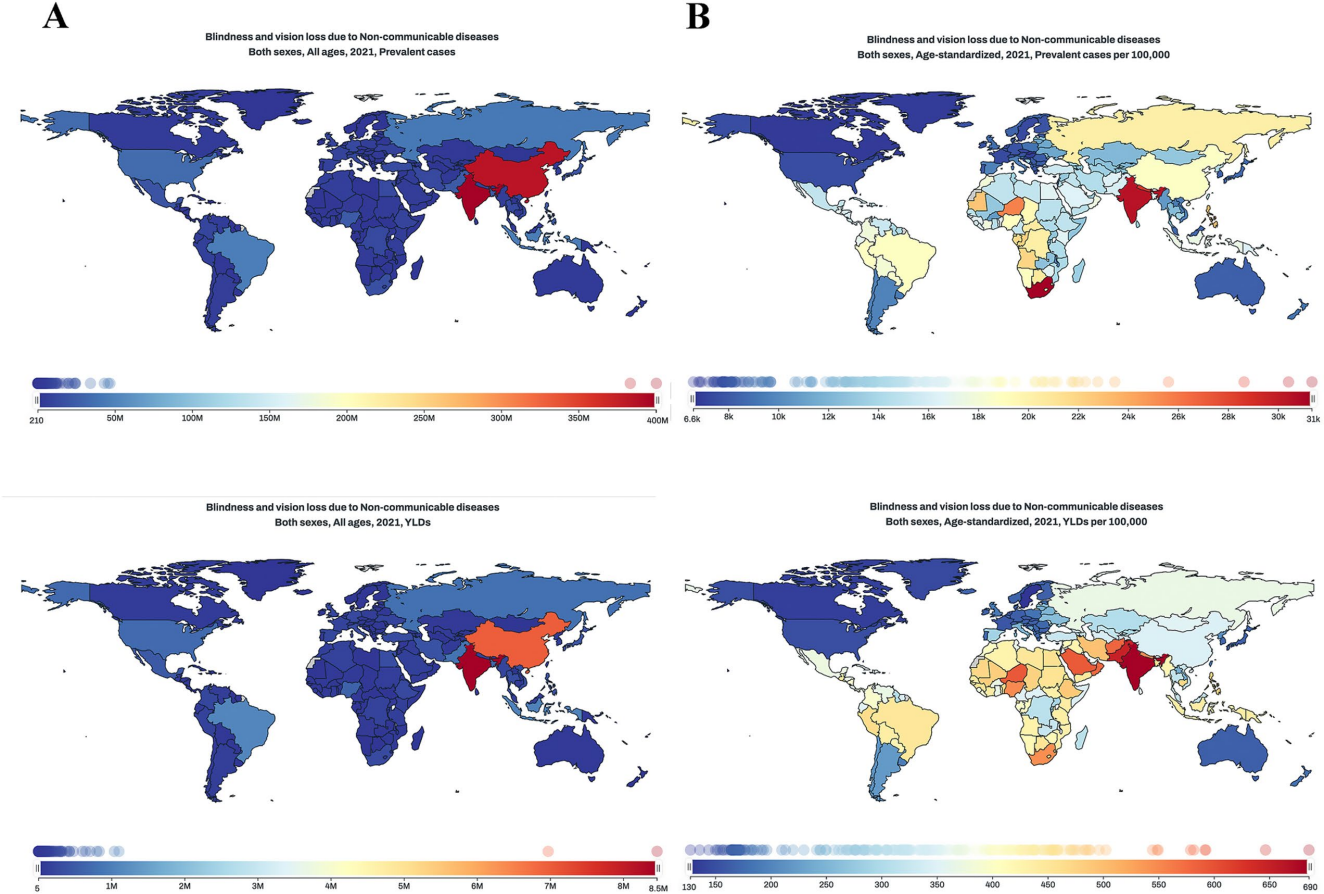
Global maps of burden of BVL caused by NCDs in 2021. **(A)** prevalence cases and ASR of BVL caused by NCDs in 2021; **(B)** YLDs number and ASR of BVL caused by NCDs in 2021. BVL, blindness and vision loss; NCDs, non-communicable diseases; ASR, age-standardized rate; YLDs, years lived with disability.

In 2021, among the 204 countries and territories, the top 3 countries with largest number of BVLs caused by NCDs were India [400198.1 (95% UI: 319256.0–491595.9) thousand], China [383330.7 (95% UI: 296595.9–493213.4) thousand], and Brazil [46674.3 (95% UI: 37553.4–58284.2) thousand], as shown in Figure 2. After standardized for age, the top 3 countries with the highest prevalence rates (per 100,000 population) were South Africa [31.3 (95% UI: 25.3–38.5) thousand], India [30.4 (95% UI: 24.6–36.8) thousand], and Nepal [28.6 (95% UI: 24.2–32.7) thousand]. In contrast, Slovakia [6.6 (95% UI:5.4–8.0)], Canada [6.8 (95% UI: 5.2–8.9)] and Greenland [6.8 (95% UI: 5.3–8.8)] had the lowest age-standardized rates (per 100,000 population).

For YLDs, India [685.5 (95% UI: 449.6–998.9)], Pakistan [646.3 (95% UI: 459.0–899.7)], and Niger [592.4 (95% UI: 406.6–859.3)] had the highest ASRs (per 100,000 population). Sweden [127.4 (95% UI: 77.6–203.0)], Canada [135.6 (95% UI: 84.2–209.0)], and Greenland [143.8 (95% UI: 90.6–218.5)] had the lowest ASRs (per 100,000 population).

### Burden of BVL caused by NCDs by SDI

**Figure 3:**
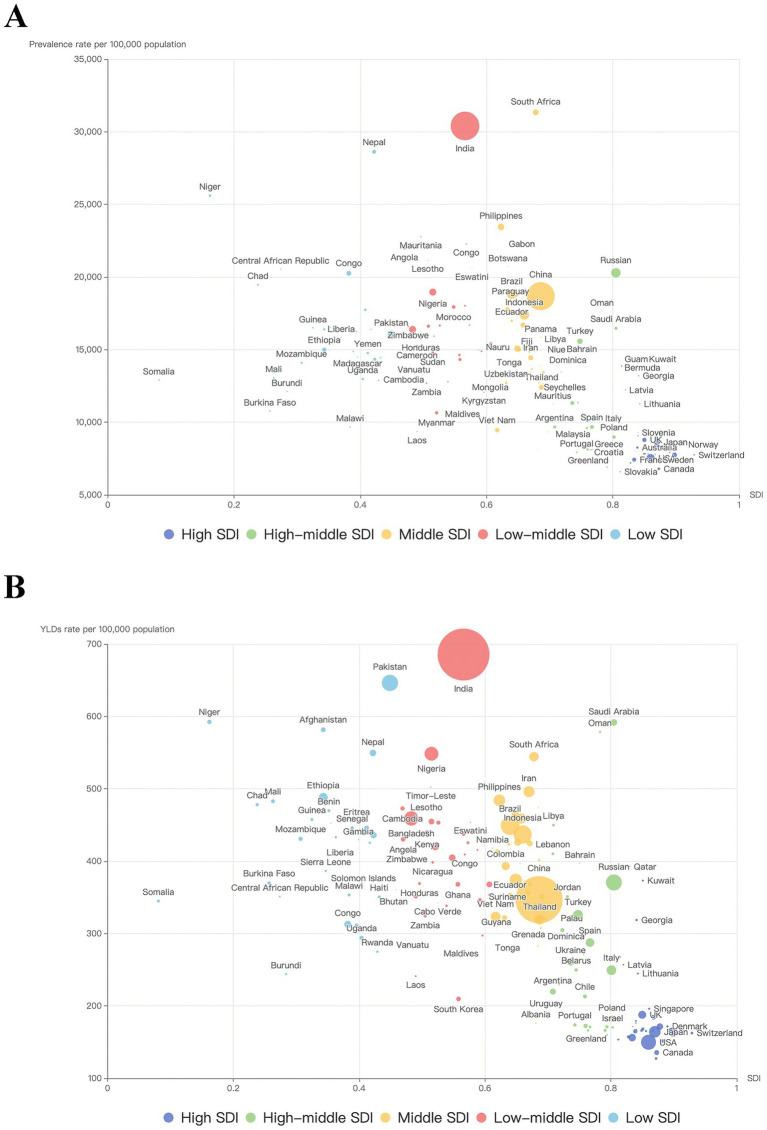
Trends in burden of BVL caused by NCDs by SDI and country in 2021. **(A)** trends in ASR of prevalence by SDI and country in 2021; **(B)** trends in ASR of YLDs by SDI and country in 2021. Each point represents a country or territory, and the diameter of each point represents the number of prevalence or YLDs. BVL, blindness and vision loss; NCDs, non-communicable diseases; ASR, age-standardized rate; SDI, Socio-demographic Index; YLDs, Years lived with disability.

**Figure 4:**
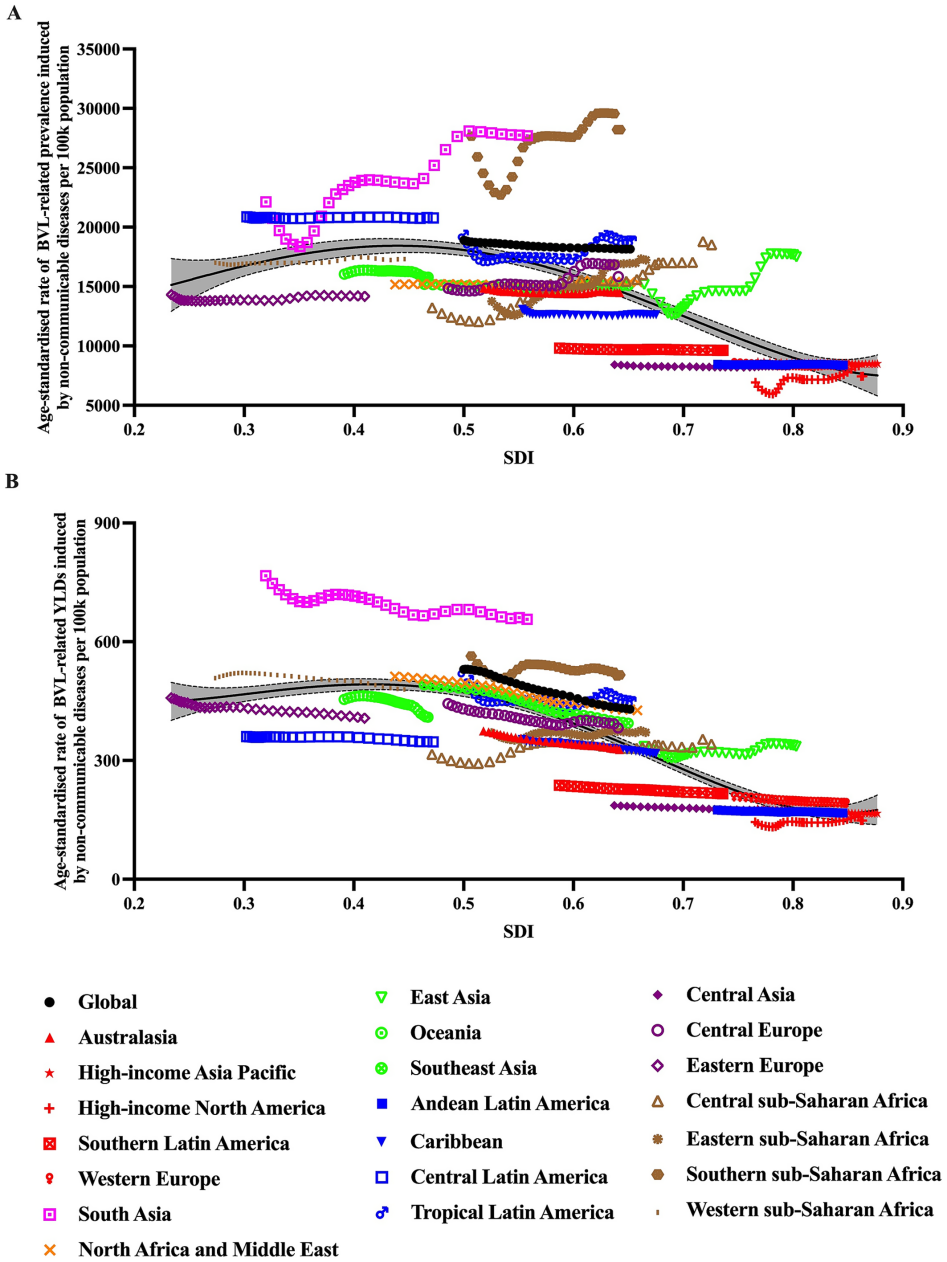
Association between burden of BVL caused by NCDs and SDI for 21 GBD super regions, 1990–2021. **(A)** association between ASR of prevalence and SDI from 1990 to 2021; **(B)** association between ASR of YLDs and SDI from 1990 to 2021. For each region, points from left to right depict estimates from each year from 1990 to 2021. BVL, blindness and vision loss; NCDs, non-communicable diseases; ASR, age-standardized rate; SDI, Socio-demographic Index; YLDs, Years lived with disability.

Figure 3 showed the burden of BVL caused by NCDs by countries or territories and SDI. As the scattergram shown, there seemed to be higher burden in those with middle SDI or lower than middle SDI. To further investigate the possible relationship between socioeconomic status and burden of BVL, Figure 4 showed the association between SDI and prevalence or YLDs of BVL caused by NCDs by region from 1990 to 2021. The ASR of burden of BVL cause by NCDs demonstrated a single-peaked relationship with the SDI level from 1990 to 2021, with the highest point appearing at low-middle SDI group.

### Burden of BVL caused by NCDs by cause and health system level

**Figure 5:**
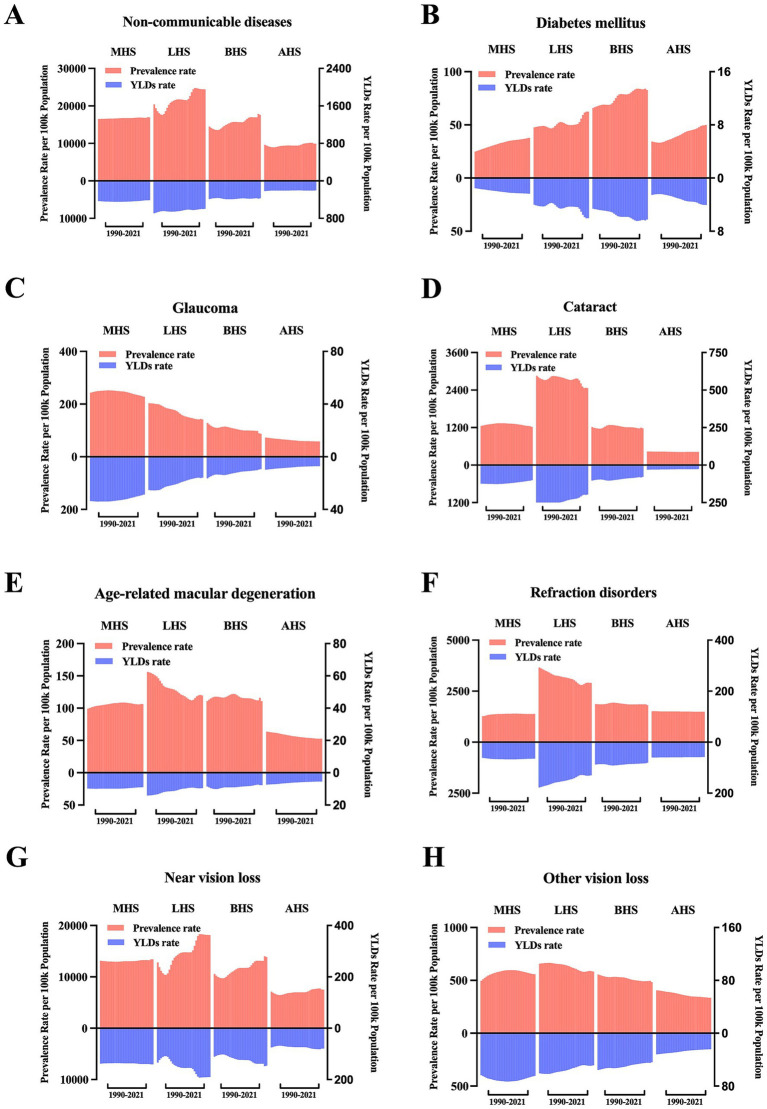
Trends in burden of BVL caused by NCDs by health system grouping level and causes. **(A)** ASR of prevalence and YLDs of BVL caused by NCDs by health system grouping level, 1990–2021; **(B)** ASR of prevalence and YLDs of BVL caused by diabetes mellitus by health system grouping level, 1990–2021; **(C)** ASR of prevalence and YLDs of BVL caused by glaucoma by health system grouping level, 1990–2021; **(D)** ASR of prevalence and YLDs of BVL caused by cataract by health system grouping level, 1990–2021; **(E)** ASR of prevalence and YLDs of BVL caused by age-related macular degeneration by health system grouping level, 1990–2021; **(F)** ASR of prevalence and YLDs of BVL caused by refraction disorders by health system grouping level, 1990–2021; **(G)** ASR of prevalence and YLDs of BVL caused by near vision loss by health system grouping level, 1990–2021; **(H)** ASR of prevalence and YLDs of BVL caused by other vision loss by health system grouping level, 1990–2021. BVL, blindness and vision loss; NCDs, non-communicable diseases; ASR, age-standardized rate; YLDs, years lived with disability; MHS, minimal health system; LHS, limited health system; BHS, basic health system; AHS, advanced health system.

The GBD study 2021 provided information on health system levels by location. As shown in Figure 5A, the overall BVL burden caused by NCDs ranked from 1990 to 2021 in each health system level subgroup. After classified according to specific causes of NCDs, subgroups including diabetes mellitus, glaucoma, cataract, age-related macular degeneration, refraction disorders, near vision loss and other vision loss (Figures 5B–H), all showed their respective changes with time and in different health system level subgroup. Noteworthily, in all health system level subgroups, BVL burden caused by diabetes mellitus and near vision loss increased with time, BVL burden caused by glaucoma decreased consistently, and burden caused by cataract remained essentially stable. Burden of refraction disorders decreased with time in low health system subgroup, but remained basically unchanged in other health system subgroups. Besides, all with time, regions with low health system were accompanied with highest burden caused by cataract, age-related macular degeneration, refraction disorders, near vision loss and other vision loss, respectively. And regions with advanced health system were accompanied with lowest burden caused by glaucoma, cataract, age-related macular degeneration, near vision loss and other vision loss, respectively.

### Future prediction of burden

On the basis of the trend observed, ARIMA model was applied to project the future trend towards 2045. As shown in Figures 1G,H, in 2045, there will be approximately 1332370.8 (95% UI: 1107580.1–1646730.0) thousand female cases and 1086769.1 (95% UI: 893705.6–1425214.0) thousand male cases. The prevalence rate per 100,000 population will reach approximately 21937.6 (95% UI: 19364.9–27043.1) in females and 19662.9 (95% UI: 15681.3–25735.4) in males. The YLDs rate will reach approximately 371.7 (95% UI: 254.2–536.0) in females and 343.1 (95% UI: 235.2–490.0) in males.

## Discussions

The current study meticulously delineates the global landscape of burden of BVL caused by NCDs across various dimensions, including year, age, sex, region, socio-economic status and health system levels from 1990 to 2021. NCDs encompass a wide array of chronic maladies, such as neoplasms, cardiovascular diseases, chronic respiratory diseases, digestive diseases, diabetes and kidney diseases, sense organ diseases, and etc. As societies progress and life expectancies lengthen, NCDs have progressively emerged as the predominant factors influencing health and disease burden. Over the past few decades, there has been an overall upward trajectory in both the global prevalence and YLDs numbers of BVL caused by NCDs. When adjusted for age and population, the ASR of prevalence has shown an increase, while the ASR of YLDs has remained largely stable. In terms of disease severity, a rising trend in presbyopia has been observed, concurrently with a decline in the prevalence of blindness. The launch of the WHO’s “VISION 2020” initiative has spurred intensified efforts to prevent blindness, achieving partial success in alleviating the disease burden associated with blindness. However, with the burgeoning aging population structure and enhanced detection rates, the escalating burden of presbyopia appears to be an almost inescapable consequence.

Regarding gender disparities, the burden of BVL had persistently been greater among females than males, a finding that aligns with prior researches (3, 13, 14). One plausible explanation is that women generally enjoy a longer life expectancy compared to men, which is often coupled with an increased susceptibility to age-related ocular conditions (15). Besides, in many countries, especially developing countries, the social norms and cultural beliefs, and poverty prevent them to come forward for medical consultation in time (16). And patients presenting to hospitals are predominantly males. This may worsen the disease condition. Additionally, it is suggested that women may face greater financial constraints in accessing spectacles for the correction of distance refractive errors and presbyopia, as well as in obtaining adequate cataract surgical coverage. To redress these extant inequities, concerted efforts must be directed towards enhancing the eye healthcare services available to women (17, 18).

Regarding age disparities, the numbers of prevalence and YLDs for BVL initially increased and then decreased. However, after adjusting for population, the rates of prevalence and YLDs continued to rise with age. This trend is primarily attributed to the specific causes of BVL caused by NCDs, which include diabetes mellitus, glaucoma, cataract, age-related macular degeneration, refraction disorders, and near vision loss, most of which are age-related conditions. Notably, the peak increase rate occurred between the ages of 35 and 40, indicating that more attention should be paid not only to the older adult but also to individuals in the 35–40 age group. This suggests that preventive measures and early interventions should be targeted at this age group to mitigate the rising burden of BVL.

Regarding regional disparities, it was not surprising that populous countries such as India and China bear the largest burden. Upon standardization, highest ASR of burden were predominantly found in underdeveloped countries, such as India, Pakistan, South Africa. Further analysis based on SDI reveals countries with low-middle or low SDI experience significantly higher burdens. The regression curve further illustrated a negative correlation between SDI and burden of BVL. In the World Health Survey, lower socioeconomic status was pinpointed as a significant risk factor for vision difficulties, with over 90% of the burden of eye diseases occurring in low-income and middle-income countries (13, 19). Further disparity analysis based on health system infrastructure robustly corroborated this observation. Generally, regions with subpar health systems bear a disproportionately higher burden. Specifically, conditions such as cataracts, age-related macular degeneration, refractive disorders, and near vision loss exhibit higher prevalence rates in areas with underdeveloped health systems. Considering the data sources of the GBD study, it is plausible that the data from countries with minimal health system may be inadequate to accurately capture the true extent of the burden, potentially leading to an underestimation in these regions. Moreover, the burden associated with the aforementioned diseases, as well as glaucoma, tends to diminish as health system levels improve. Notably, the BVL burden of glaucoma also demonstrated a significant downward trajectory over time. However, it is crucial to highlight that the BVL burden linked to diabetes mellitus has persistently increased over time and in tandem with the level of health systems.

Cataract, recognized as the most common cause of blindness, had been previously identified as inversely correlated with socioeconomic status (20). A possible explanation for this correlation is that the level of the SDI is intricately linked to both the output and quality of cataract surgery. Cataract surgery is widely regarded as one of the most cost-effective health interventions, and its frequency is on the rise across all regions. A systematic review has highlighted that there exists inequality in cataract surgery rates among countries categorized by income levels, with this disparity being associated with various socioeconomic indicators (21). Glaucoma, the second most prevalent cause of blindness, has also been reported to exhibit an unequal distribution of its burden (22). This inequality may potentially be attributed to factors such as prolonged exposure to ultraviolet radiation and limited access to medical care (23). Similarly, age-related macular degeneration has been previously found to follow a pattern of inequality in its burden distribution (24). Regarding BVL caused by diabetes mellitus, the highest burden is observed in regions with basic health systems. In line with this, our previous research has also described a higher burden attributable to high fasting plasma glucose in regions with middle SDI (25), and a higher burden caused by diabetic retinopathy in regions with high-middle SDI (26). There is no doubt that a higher socioeconomic level, coupled with better-equipped healthcare systems, can significantly enhance access to preventative medicine. This includes a range of services vital for early detection, early diagnosis, and early treatment of diseases, thereby playing a crucial role in mitigating the overall burden of BVL (27). Countries with low SDI are facing a dual challenge: they contend with a broad spectrum of diseases while also grappling with limited human resources and poor accessibility to specialized tertiary eye-care services (28).

Our study offered a comprehensive understanding of the burden of BVL caused by NCDs and the disparities that exist across multiple dimensions. However, it is important to acknowledge several limitations inherent in our approach. Firstly, our analysis focused on global, regional, and national epidemiological trends, which may overlook micro-level variations, particularly in countries with large populations. To gain a more nuanced understanding, sub-national analyses are necessary to accurately estimate the BVL burden attributable to NCDs, and future research should be directed towards specific areas where such detailed assessments are lacking. Secondly, our study relied on data from the GBD study. The data estimates within the GBD study are derived from a diverse array of data collection methods and sources. In less-developed countries, data collection may be either absent or limited, leading to potential heterogeneity and impacting the overall data quality. This variability in data sources and collection methods could introduce uncertainties in our findings. Thirdly, given that the data in the GBD Study are updated periodically, the results of our current analysis may not remain applicable in the near future. As new data becomes available and methodologies evolve, the landscape of BVL burden and its associated disparities may shift, necessitating ongoing research and updates to our understanding.

## Conclusion

In conclusion, this study revealed the global health burden of BVL caused by NCDs from 1990 to 2021, dissecting the data by region, sex, age, SDI, health system levels and specific causes. As we project into the future, even up to 2045, BVL caused by NCDs is poised to remain a pressing public health concern, with disparities persisting across different regions, genders, and age groups. Healthcare programs that target BVL must become increasingly imperative, especially for females, the older adult, regions with lower SDI or less advanced health systems. Policies support for blindness early screening, preferential policies for females and the older adult and strengthening healthcare systems in vulnerable regions, such as more comprehensive insurance coverage, incorporating eye health into primary care settings or routine check-ups, emphasizing preventive care and co-morbid conditions management for older adults, raising public awareness and enhancing public health capacity, if effectively implemented, can help reduce disparities in vision and eye health. We hope that this study can prove instrumental in informing policy decisions and guiding the allocation of resources to effectively address this significant health challenge.

## Data Availability

The original contributions presented in the study are included in the article/Supplementary material, further inquiries can be directed to the corresponding author.

## Footnotes

^1^ https://vizhub.healthdata.org/gbd-results/

## References

[1] Wang L Zhu Z Scheetz J He M. Visual impairment and ten-year mortality: the Liwan eye study. Eye (Lond). (2021) 35:2173–9. doi: 10.1038/s41433-020-01226-x, PMID: 33077908 PMC8302561

[2] Marques AP Ramke J Cairns J Butt T Zhang JH Muirhead D. Global economic productivity losses from vision impairment and blindness. EClinicalMedicine. (2021) 35:100852. doi: 10.1016/j.eclinm.2021.100852, PMID: 33997744 PMC8093883

[3] Bourne R Steinmetz JD Flaxman S Briant PS Taylor HR Resnikoff S. Trends in prevalence of blindness and distance and near vision impairment over 30 years: an analysis for the global burden of disease study. Lancet Glob Health. (2021) 9:e130–43. doi: 10.1016/S2214-109X(20)30425-3, PMID: 33275950 PMC7820390

[4] Pizzarello L Abiose A Ffytche T Duerksen R Thulasiraj R Taylor H. Vision 2020: the right to sight: a global initiative to eliminate avoidable blindness. Arch Ophthalmol. (2004) 122:615–20. doi: 10.1001/archopht.122.4.615, PMID: 15078680

[5] Zhou M Wang H Zeng X Yin P Zhu J Chen W. Mortality, morbidity, and risk factors in China and its provinces, 1990-2017: a systematic analysis for the global burden of disease study 2017. Lancet. (2019) 394:1145–58. doi: 10.1016/S0140-6736(19)30427-1, PMID: 31248666 PMC6891889

[6] Wong TY Zheng Y Jonas JB Flaxman SR Keeffe J Leasher J. Prevalence and causes of vision loss in East Asia: 1990-2010. Br J Ophthalmol. (2014) 98:599–604. doi: 10.1136/bjophthalmol-2013-304047, PMID: 24390167

[7] WHO. Noncommunicable diseases. [EB/OL]. (2024-12-23)[2025-03-04]. Available online at: https://www.who.int/news-room/fact-sheets/detail/noncommunicable-diseases (Accessed March 4, 2025).

[8] Omran AR. The epidemiologic transition. A theory of the epidemiology of population change. Milbank Mem Fund Q. (1971) 49:509–38. doi: 10.2307/3349375, PMID: 5155251

[9] Steinmetz JD Bourne RR Briant PS Flaxman SR Taylor HR Jonas JB. Causes of blindness and vision impairment in 2020 and trends over 30 years, and prevalence of avoidable blindness in relation to VISION 2020: the right to sight: an analysis for the global burden of disease study. Lancet Glob Health. (2021) 9:e144–60. doi: 10.1016/S2214-109X(20)30489-7, PMID: 33275949 PMC7820391

[10] Ferrari AJ Santomauro DF Aali A Abate YH Abbafati C Abbastabar H. Global incidence, prevalence, years lived with disability (YLDs), disability-adjusted life-years (DALYs), and healthy life expectancy (HALE) for 371 diseases and injuries in 204 countries and territories and 811 subnational locations, 1990-2021: a systematic analysis for the global burden of disease study 2021. Lancet. (2024) 403:2133–61. doi: 10.1016/S0140-6736(24)00757-8, PMID: 38642570 PMC11122111

[11] Foreman KJ Marquez N Dolgert A Fukutaki K Fullman N McGaughey M. Forecasting life expectancy, years of life lost, and all-cause and cause-specific mortality for 250 causes of death: reference and alternative scenarios for 2016-40 for 195 countries and territories. Lancet. (2018) 392:2052–90. doi: 10.1016/S0140-6736(18)31694-5, PMID: 30340847 PMC6227505

[12] Longtin Y Paquet-Bolduc B Gilca R Garenc C Fortin E Longtin J. Effect of detecting and isolating *Clostridium difficile* carriers at hospital admission on the incidence of C difficile infections: a quasi-experimental controlled study. JAMA Intern Med. (2016) 176:796–804. doi: 10.1001/jamainternmed.2016.0177, PMID: 27111806

[13] Ono K Hiratsuka Y Murakami A. Global inequality in eye health: country-level analysis from the global burden of disease study. Am J Public Health. (2010) 100:1784–8. doi: 10.2105/AJPH.2009.187930, PMID: 20634443 PMC2920965

[14] Doyal L Das-Bhaumik RG. Sex, gender and blindness: a new framework for equity. BMJ Open Ophthalmol. (2018) 3:e000135. doi: 10.1136/bmjophth-2017-000135, PMID: 30246151 PMC6146307

[15] Abou-Gareeb I Lewallen S Bassett K Courtright P. Gender and blindness: a meta-analysis of population-based prevalence surveys. Ophthalmic Epidemiol. (2001) 8:39–56. doi: 10.1076/opep.8.1.39.1540, PMID: 11262681

[16] Di Cesare M Khang YH Asaria P Blakely T Cowan MJ Farzadfar F. Inequalities in non-communicable diseases and effective responses. Lancet. (2013) 381:585–97. doi: 10.1016/S0140-6736(12)61851-0, PMID: 23410608

[17] Lewallen S Mousa A Bassett K Courtright P. Cataract surgical coverage remains lower in women. Br J Ophthalmol. (2009) 93:295–8. doi: 10.1136/bjo.2008.140301, PMID: 19091848

[18] Ye Q Chen Y Yan W Wang W Zhong J Tang C. Female gender remains a significant barrier to access cataract surgery in South Asia: a systematic review and Meta-analysis. J Ophthalmol. (2020) 2020:1–14. doi: 10.1155/2020/2091462, PMID: 32411426 PMC7201788

[19] Freeman EE Roy-Gagnon MH Samson E Haddad S Aubin MJ Vela C. The global burden of visual difficulty in low, middle, and high income countries. PLoS One. (2013) 8:e63315. doi: 10.1371/journal.pone.0063315, PMID: 23675477 PMC3651198

[20] Deng Y Yang D Yu JM Xu JX Hua H Chen RT. The association of socioeconomic status with the burden of cataract-related blindness and the effect of ultraviolet radiation exposure: an ecological study. Biomed Environ Sci. (2021) 34:101–9. doi: 10.3967/bes2021.015, PMID: 33685568

[21] Yan W Wang W van Wijngaarden P Mueller A He M. Longitudinal changes in global cataract surgery rate inequality and associations with socioeconomic indices. Clin Experiment Ophthalmol. (2019) 47:453–60. doi: 10.1111/ceo.13430, PMID: 30362287

[22] Chen W Xu Y Liu Z Zhao J. Global, regional and national burden of Glaucoma: an update analysis from the global burden of disease study 2019. Int Ophthalmol. (2024) 44:234. doi: 10.1007/s10792-024-03222-6, PMID: 38896279

[23] Dai J Suo L Xian H Pan Z Zhang C. Investigating the impact of sun/UV protection and ease of skin tanning on the risk of Pseudoexfoliation Glaucoma: a Mendelian randomization study. Invest Ophthalmol Vis Sci. (2023) 64:4. doi: 10.1167/iovs.64.13.4, PMID: 37788000 PMC10552876

[24] Jiang B Jiang C Li J Lu P. Trends and disparities in disease burden of age-related macular degeneration from 1990 to 2019: results from the global burden of disease study 2019. Front Public Health. (2023) 11:1138428. doi: 10.3389/fpubh.2023.1138428, PMID: 37265519 PMC10231224

[25] Ye L Xu J Zhang T Lin X Pan X Zeng W. Global burden of noncommunicable diseases attributable to high fasting plasma glucose. J Diabetes. (2020) 12:807–18. doi: 10.1111/1753-0407.13072, PMID: 32472661

[26] Shan Y Xu Y Ye L Lin X Chen Y Miao Q. Socioeconomic disparity in global vision loss burden due to diabetic retinopathy: an analysis on time trends from 1990 to 2017. Endocrine. (2021) 73:316–24. doi: 10.1007/s12020-021-02692-3, PMID: 34101111

[27] Xu X Wu J Yu X Tang Y Tang X Shentu X. Regional differences in the global burden of age-related macular degeneration. BMC Public Health. (2020) 20:410. doi: 10.1186/s12889-020-8445-y, PMID: 32228540 PMC7106756

[28] Rai BB Morley MG Bernstein PS Maddess T. Pattern of vitreo-retinal diseases at the national referral hospital in Bhutan: a retrospective, hospital-based study. BMC Ophthalmol. (2020) 20:51. doi: 10.1186/s12886-020-01335-x, PMID: 32054472 PMC7017569

